# Does T-tube indwelling prolong the procedure of endoscopic retrograde cholangiopancreatography for healing duct-to-duct anastomotic bile leakage after liver transplantation?

**DOI:** 10.1097/MD.0000000000040191

**Published:** 2024-10-25

**Authors:** Songming Ding, Shanjie Dong, Hengkai Zhu, Shusen Zheng, Qiyong Li

**Affiliations:** a Division of Hepatobiliary and Pancreatic Surgery, Shulan (Hangzhou) Hospital Affiliated to Zhejiang Shuren University, Shulan International Medical College, Hangzhou, Zhejiang, P.R. China.

**Keywords:** bile leakage, duct-to-duct anastomosis, endoscopic retrograde cholangiopancreatography, liver transplantation, T-tube

## Abstract

Endoscopic retrograde cholangiopancreatography (ERCP) is the preferred treatment for duct-to-duct anastomotic bile leakage (D-D aBL) after liver transplantation (LT). This study aimed to compare the time required for ERCP and D-D aBL recovery in post-LT patients with and without T-tube drainage. A total of 40 patients (11 with T-tube drainage and 29 without T-tube drainage) with confirmed D-D aBLs treated successfully with ERCP from July 2016 to September 2021 were reviewed. The mean interval from LT to initial ERCP was significantly longer in patients with T-tube drainage than in those without T-tube drainage (41.9 vs 25.1 days, *P* < .05). However, there was no significant difference in the time required for ERCP to result in D-D aBL healing between patients with T-tube drainage and those without T-tubes (33.4 vs 23.0 days). T-tube indwelling did not significantly prolong the course required for ERCP to resolve D-D aBL in post-LT patients.

## 1. Introduction

Biliary complications, such as the technical Achilles heel of liver transplantation (LT),^[1]^ have an incidence rate ranging from 5% to 45% and account for significant morbidity and mortality.^[2–6]^ Biliary complications after LT include biliary tract strictures, bile leakages (BLs), bile duct stones, bile casts, cholangitis, and hemobilia.^[7]^

BL, a secondary adverse event with a prevalence of 2% to 25%,^[8]^ is the most common cause of death related to the biliary tract after LT.^[9]^ BLs are classified as anastomotic, T-tube removal-related, cystic duct-related, or cut surface-related leaks.^[10]^ Multiple risk factors for BL have been identified, including technical factors during surgery, donor LT after cardiac death, hepatic artery ischemia, ABO incompatibility, and prolonged cold ischemic periods.^[8]^

Patients with BL are managed with surgery, percutaneous transhepatic biliary drainage, and endoscopic retrograde cholangiopancreatography (ERCP). Surgical repair has a higher mortality rate, and percutaneous transhepatic biliary drainage has a lower clinical success rate due to the lack of significant dilation of the intrahepatic bile duct.^[11]^ ERCP-guided endoscopic therapy has emerged as the preferred intervention for duct-to-duct anastomotic bile leakage (D-D aBL) in patients after LT due to its high-resolution rates and safety.^[1,12]^

T-tube insertion is controversial during D-D biliary reconstruction procedures in LT. Some centers prefer the placement of T-tubes as a prophylactic method for anastomotic strictures.^[1,7]^ Several comparative studies have indicated that T-tubes are a risk factor for the development of BL.^[13,14]^ Traditionally, BL developed at the T-tube insertion site was self-limiting and could be managed conservatively.^[2]^ However, if persistent extrahepatic bile collection, fluctuations in liver function indices, and patients have a fever, abdominal pain, or any sign of bile-related peritonitis, endoscopic treatment is recommended. T-tube tract maturation is delayed in patients after LT because of immunosuppression. This study aimed to compare the time required for ERCP treatment to heal D-D aBL in post-LT patients with and without T-tube drainage.

## 2. Materials and methods

### 2.1. Patients

This retrospective descriptive study was approved by the Ethics and Institutional Review Board of Shulan Hospital (Hangzhou), which is affiliated with Shulan International Medical College, Zhejiang Shuren University, Hangzhou, Zhejiang, PR China, from July 2016 to September 2021 (Reference Number: KY2021057). BL was confirmed by leakage of contrast medium from the bile duct anastomosis into the peritoneal cavity during ERCP. All enrolled patients presented with no decreasing trend in the presence of bile-like drainage or clinical symptoms, such as fever, abdominal pain, or any sign of bile-related peritonitis. The inclusion criteria were as follows: patients with LT and ERCP procedures for BLs performed at our hospital and the age of the patients ≥18 years. The exclusion criteria consisted of patients exhibiting BL combined with rejection (confirmed by liver puncture pathology), BL combined with ischemic cholangiopathy, cystic duct-related BL, or cut surface-related BL after partial LT, and patients who died within 3 months after LT. BL resolution was defined as the cessation of bile-like drain output and/or the absence of contrast extravasation on subsequent ERCP after the initial endotherapy or T-tube cholangiography.^[8]^ Anastomotic stricture was defined as an abrupt narrowing of the lumen with proximal donor bile duct dilation.^[12]^ Follow-up time should be at least 3 months after LT.

### 2.2. Endoscopic procedure

ERCP was performed by professional endoscopists under moderate and deep sedation without endotracheal intubation using an Olympus Duodenoscope (JF-240/TJF-260; Olympus Optical Co., Ltd., Tokyo, Japan). For BL within 2 weeks of LT, our center usually uses endoscopic nasobiliary drainage (ENBD) with or without sphincterotomy as the initial intervention. A biliary stent was placed if BL combined with an anastomotic stricture was detected. Plastic stents were used if the bile duct diameter was <5 to 6 mm; otherwise, metal stents were used. Plastic or metal stents can be placed for up to 2 to 3 months. Stents were removed, and ENBDs were used in patients presenting with cholangitis after stent implantation. Blood amylase levels were routinely measured after ERCP. To prevent post-ERCP pancreatitis, a pancreatic stent was placed when the guidewire entered the pancreatic duct more than 3 times. For abdominal pain combined with elevated amylase levels after ERCP, the administration of pharmacological agents (such as somatostatin or octreotide) was scheduled. All patients received intravenous broad-spectrum antibiotics adjusted according to the results of the drug sensitivity analysis. The use of immunosuppressive therapy and the monitoring of drug concentrations in all patients met the treatment standards.^[15,16]^

### 2.3. Statistical analysis

Chi-square or Fisher exact test was used for categorical variables, and Student *t* test or Mann–Whitney *U* test was used for continuous variables. A *P* value <.05 was considered statistically significant. All analyses were performed using IBM SPSS for Windows version 19.0 (IBM, Armonk, NY).

## 3. Results

This study enrolled 40 patients. The basic characteristics of the enrolled patients are summarized in Table 1. The patients with T-tubes consisted of 10 men and 1 woman with a mean age of 46.9 years (range, 31–68 years). The patients without T-tubes consisted of 25 men and 4 women with a mean age of 51.8 years (range, 33–68 years). The 2 groups had no significant difference in body mass index (22.8 vs 22.7 kg/m^2^). The proportion of patients with ABO incompatibility in the T-tube drainage group was slightly higher than that in the group without T-tube drainage (18.2% vs 3.4%). The proportion of patients with continuous suturing of the posterior wall and intermittent anastomosis of the anterior wall was significantly higher in the T-tube group than in the non-T-tube group (90.9% vs 41.4%, *P* < .05).

**Table 1:** Baseline Characteristics of the 40 Patients.

Variables	T-tube group	Without T-tube group
Mean age (range), yr	46.9 (31–68)	51.8 (33–68)
Sex, male (%)	90.9	86.2
Diabetes mellitus, yes (%)	27.3	17.2
Hypertension, yes (%)	27.3	17.2
Mean BMI (range), kg/m^2^	22.8 (16.6–30.1)	22.7 (17.0–28.7)
Underlying disease, malignancy (%)	54.5	44.8
Blood type (A/B/AB/O), (%)	27.3/9.1/18.2/45.5	27.6/24.1/10.3/37.9
Blood group incompatibility, yes (%)	18.2	3.4
Anastomotic mode of bile duct* (1/2), (%)	9.1/90.9	58.6/41.4
Highest ratio of drainage TB to serum TB pre-ERCP (range)	17.2 (1.0–59.1)	20.9 (0.6–59.2)
Mean pre-initial ERCP ALB (range), g/L	35.6 (30.1–50.0)	36.8 (28.4–50.3)
Mean pre-initial ERCP ALT (range), U/L	73.2 (13.0–344.0)	59.9 (6.0–279.0)
Mean pre-initial ERCP AST (range), U/L	88.3 (43.0–276.0)	68.4 (18.0–188.0)
Mean pre-initial ERCP GGT (range), U/L	208.3 (10.0–1035.0)	94.1 (13.0–230.0)
Mean pre-initial ERCP AKP (range), U/L	262.6 (98.0–1132.0)	137.9 (61.0–301.0)
Mean pre-initial ERCP TB (range), µmol/L	208.7 (19.0–594.0)	180.9 (4.0–729.0)
Mean pre-initial ERCP DB (range), µmol/L	134.7 (11.0–380.0)	137.5 (2.0–604.0)
Mean pre-initial ERCP IB (range), µmol/L	74.0 (8.0–252.0)	41.8 (2.0–125.0)

* There was a statistical difference between the 2 groups; BMI = body mass index (weight/height^2^); Bile duct anastomotic mode 1: intermittent anastomosis of the anterior and posterior wall; Bile duct anastomotic mode 2: continuous suture of the posterior wall and intermittent anastomosis of the anterior wall.

AKP = alkaline phosphatase, ALB = albumin, ALT = alanine aminotransferase, AST = aspartate aminotransferase, DB = direct bilirubin, ERCP = endoscopic retrograde cholangiopancreatography, GGT = γ-glutamyl transferase, IB = indirect bilirubin, LT = liver transplantation, TB = total bilirubin.

Endoscopic treatments and findings are shown in Table 2. The mean number of ERCP procedures in the group with T-tube drainage was 1.7 (range, 1–3), and that in the group without T-tube drainage was 1.6 (range, 1–4). The proportion of patients who underwent endoscopic sphincterotomy with T-tube drainage was slightly higher than that without T-tube drainage (54.5% vs 27.6%). It should be emphasized that sphincterotomy in this study was not solely for treating D-D aBL but was mainly related to the difficulty of intubation. In the T-tube group, the proportions of patients receiving ENBD treatment, biliary stent placement, and ENBD combined with biliary stent placement were 27.3%, 27.3%, and 45.5%, respectively. In the without T-tube group, the proportions of patients receiving ENBD treatment, stent placement, and ENBD combined with stent placement were 48.3%, 6.9%, and 44.8%, respectively. The proportion of patients who underwent initial ERCP within 4 weeks after LT was lower in the group with T-tube drainage than in the group without T-tube drainage (45.5% vs 62.1%). The mean interval from LT to initial ERCP in the group with T-tube drainage was significantly longer than in the group without T-tube drainage (41.9 vs 25.1 days, *P* < .05). The hospital stay from LT to complete resolution of D-D aBL in the group with T-tube drainage was also longer than in the group without T-tube drainage (75.3 vs 48.1 days, *P* < .05). However, there was no significant difference in the time from the initial ERCP to complete BL healing between the groups with and without T-tube drainage (33.4 vs 23.0 days).

**Table 2:** Endotherapy procedure and endoscopic finding.

Variables	T-tube group	Without T-tube group
Sphincterotomy, yes (%)	54.5	27.6
Mean number of ERCP (range)	1.7 (1–3)	1.6 (1–4)
Endoscopic treatment mode for bile leakage (1/2/3), yes (%)	27.3/27.3/45.5	48.3/6.9/44.8
Initial ERCP within 4 wk after LT, yes (%)	45.5	62.1
Mean interval from LT to initial ERCP (range)*, d	41.9 (20–132)	25.1 (4–60)
Mean time from LT to bile leakage cessation (range)*, d	75.3 (45–150)	48.1(15–81)
Mean time from initial ERCP to bile leakage cessation (range), d	33.4 (5–87)	23.0 (7–47)

* There was a statistical difference between the 2 groups; endoscopic treatment mode 1: endoscopic nasobiliary drainage; endoscopic treatment mode 2: biliary stent placement; endoscopic treatment mode 3: endoscopic nasobiliary drainage subsequent biliary stent placement.

ERCP = endoscopic retrograde cholangiopancreatography, LT = liver transplantation.

No obvious adverse events associated with ERCP, such as gastrointestinal tract perforation, severe pancreatitis, or intraabdominal bleeding, have been reported. In all patients, the indwelling T-tubes were removed, and there were no complications such as biliary peritonitis after T-tube extraction.

## 4. Discussion

BL, the second most common biliary complication after LT, can be asymptomatic or present with a clinical picture of abdominal infection, peritonitis, and graft dysfunction.^[17–19]^ Patients with BLs have a relatively poor long-term prognosis, and the 1-year survival rate for patients with BLs is 76% versus 89% for those without BLs.^[20]^ BL can be derived from duct-to-duct anastomosis (with or without a T-tube), the cut surface of a split liver, cystic duct remnants, and accessory ducts in the gallbladder fossa.^[21,22]^ BLs can be divided into early (within 4 weeks of LT) and late leaks.^[7,10]^ Early leaks are usually related to an insufficient local blood supply to the bile duct due to thrombosis of the liver artery and technical concerns such as suture skills and experience of the operator.^[7,18]^ Late leaks can be due to T-tube removal, migration, and perforation of the biliary stent or late-onset ischemia of the liver artery.^[18,23]^

It has been claimed that BLs with T-tube are more likely to occur later than without T-tube drainage.^[12]^ We found that the proportion of patients who underwent initial ERCP within 4 weeks after LT was lower in the T-tube group than in the control group (45.5% vs 62.1%). Moreover, the mean time from LT to the initial ERCP in the group with the T-tube was significantly longer than in the group without the T-tube drainage (41.9 vs 25.1 days).

ERCP is one of the first options for BL in patients after LT and has a high-resolution rate and safety profile.^[24,25]^ BLs can be managed by ENBD,^[26]^ endoscopic sphincterotomy,^[27]^ placement of a plastic biliary stent and/or a fully covered self-expandable metallic stent with or without endoscopic sphincterotomy, or by a combination of these procedures.^[28–32]^ Currently, endoscopic sphincterotomy alone is not recommended for the treatment of BL after LT. Effective ENBD may resolve all BL.^[33]^ Generally, ENBD, with or without sphincterotomy, is the first treatment choice at our center. If patients cannot tolerate the discomfort caused by an ENBD tube or BL combined with an anastomotic stricture, biliary stent placement is a good option. It has been reported that the biliary stent placement can heal 88% of BLs.^[9]^ Fully covered self-expandable metallic stents have recently been used to treat refractory BLs.^[34]^ To achieve a high clinical success rate, the median time for placement of ENBD tube was 9 days (range, 2–81 days).^[12]^ Usually, biliary stent patency should be maintained for up to 2 to 3 months to ensure proper healing of the leaks.^[35]^

In clinical practice, the timing of endoscopic treatment of BL is the most challenging. The onset of BL may range from 1 day to 6 months after LT.^[36]^ Endoscopic treatment for BL on the first day after LT is a high-risk procedure. Furthermore, one-third to one-half of the BLs developed at the T-tube insertion site closed spontaneously within 24 hours.^[6]^ Thus, such BLs can be treated by “wait and see.” However, some patients with persistent BLs required endotherapy after conservative observation. In our center, the indications for endoscopic treatment include no decreasing trend in the presence of bile-like drainage or clinical symptoms such as fever, abdominal pain, or any sign of bile-related peritonitis.

This study aimed to explore whether the time required for ERCP to seal the BL in post-LT patients with T-tubes was longer than in patients without T-tube drainage. There were no significant differences in the liver function indices before ERCP, age, body mass index, ratio of bilirubin from the drainage fluid to serum bilirubin, sex composition, blood type distribution, proportion of malignant disease, or proportion of ABO incompatibility between the 2 groups. The proportion of patients with continuous suturing of the posterior wall and intermittent anastomosis of the anterior wall among those with T-tube drainage was significantly higher than in those without T-tube drainage (90.9% vs 41.4%). We speculated that the anastomosis mode of the bile duct could influence the treatment of BL. However, this did not affect the healing of the bile duct. Finally, we found no significant difference in the time from initial ERCP to complete BL healing between the groups with and without T-tube drainage (33.4 vs 23.0 days).

Notably, the stent-associated cholangitis was effectively controlled after treatment with ENBD and broad-spectrum antibiotics. The limitation of this study is its retrospective design. In addition, the number of patients included in the study may have been insufficient, and the difference in the number of patients between the 2 groups may have influenced the statistical results.

In conclusion, data from our single medical center showed that indwelling T-tubes did not significantly affect the course required for ERCP to resolve D-D aBL in post-LT patients. In future studies, more patients should be included to investigate factors related to the cure of D-D aBL.

## Author contributions

**Conceptualization:** Songming Ding, Shusen Zheng.

**Data curation:** Songming Ding, Shanjie Dong.

**Writing – original draft:** Songming Ding, Qiyong Li.

**Formal analysis:** Shanjie Dong, Hengkai Zhu.

**Investigation:** Shanjie Dong, Hengkai Zhu.

**Writing – review & editing:** Shusen Zheng, Qiyong Li.
